# Magnetic resonance imaging features of bile duct adenoma

**DOI:** 10.3389/fonc.2023.1180186

**Published:** 2023-08-16

**Authors:** Mengyue Huang, Mengna Huang, Xuemei Gao, Yong Zhang, Jingliang Cheng, Jinxia Zhu, Caixia Li, Jingjing Liu

**Affiliations:** ^1^ Department of Magnetic Resonance Imaging, The First Affiliated Hospital of Zhengzhou University, Zhengzhou, China; ^2^ Magnetic Resonance Imaging (MRI) Collaboration, Siemens Healthcare Ltd., Beijing, China; ^3^ Department of Magnetic Resonance Imaging, the First Affiliated Hospital of Henan Polytechnic University, Jiaozuo, Henan, China

**Keywords:** adenoma, bile duct, malignancy, pathology, magnetic resonance imaging

## Abstract

**Objectives:**

To evaluate the magnetic resonance imaging (MRI) features of bile duct adenoma.

**Methods:**

The data of 28 patients [with 32 pathologically confirmed bile duct adenomas, including 15 with malignant change (malignant group) and 17 without malignant change (benign adenoma group)] were retrospectively reviewed. Abdominal MRI was performed for all patients; in addition, dynamic enhanced MRI was performed for 18 lesions. The MRI features, including lesion location, maximum size, morphology, signal characteristics, enhancement type, and appearance of the bile duct, were assessed by two abdominal radiologists. Apparent diffusion coefficient (ADC) values were measured and compared.

**Results:**

Of the 32 bile duct adenomas, 22 (68.75%) involved the common bile duct (CBD). While 14/32 (43.75%) lesions presented as focal eccentric-type masses, 9/32 (28.13%) presented as plaque-like masses, 4/32 (12.50%) as bile duct casting masses, and 5/32 (15.62%) as infiltrative masses. A frond-like superficial appearance was seen in 8/32 (25%) lesions. Infiltrative masses were significantly more common in the malignant group than in the benign adenoma group (*P* = 0.015). While 23/32 (71.88%) lesions were isointense on T1-weighted imaging (T1WI), 24/32 (75%) were hyperintense on T2-weighted imaging (T2WI). Bile duct dilatation was present upstream of the lesion in all cases. Bile duct dilatation at the lesion was seen in 24/32 (75%) cases and downstream of the lesion in 6/32 (18.75%) cases. Of the 18 lesions that underwent dynamic enhanced MRI, 14 (77.78%) showed moderate enhancement and 13 (72.22%) showed persistent enhancement. On diffusion-weighted imaging (DWI), 27/32 (84.37%) lesions showed hyperintensity. Mean ADC value was comparable between the malignant group and the benign adenoma group (*P =* 0.156).

**Conclusions:**

Bile duct adenoma primarily presents as intraductal growth in the CBD, usually with bile duct dilatation at the lesion site or upstream to it. Most lesions are isointense on T1WI, are hyperintense on T2WI and DWI, and show moderate enhancement. A superficial frond-like appearance of the lesion and bile duct dilatation at the lesion or downstream to it might be characteristics of bile duct adenoma. An infiltrative appearance might indicate malignant transformation.

## Introduction

Benign tumors of the bile duct are rarer than malignant ones. Bile duct adenomas, first reported by Carrolli in 1959, account for two-thirds of all benign biliary tumors (1) and for 0.1% of all biliary tract surgeries (2). Bile duct adenomas arise from the bile duct epithelium. The incidence and pathogenesis remain unclear (3). Because of the risk of recurrence and progression to cholangiocarcinoma, curative resection is the treatment of choice for bile duct adenomas (4). However, preoperative diagnosis is difficult because clinical symptoms and laboratory tests are nonspecific (5). Definitive diagnosis requires pathological examination, which is invasive, and the materials used for pathological puncture are limited (6).

Magnetic resonance imaging (MRI), with its high soft-tissue resolution, is an important noninvasive modality for the diagnosis of hepatobiliary disease (7), but the MRI characteristics of bile duct adenoma have not yet been clearly defined. In the report on bile duct adenoma by Aslam et al. (8), the pictures show hypointensity on the T1-weighted image (T1WI) and hyperintensity on the T2-weighted image (T2WI), but the authors did not describe in detail the signal characteristics of bile duct adenomas.

The aim of this retrospective study was to analyze the MRI features of bile duct adenomas diagnosed at our center.

## Methods

This cross-sectional retrospective study was approved by our hospital’s institutional review board, with a waiver of the need for informed consent.

### Patients

The study sample was selected by searching our institution’s pathology database to identify all patients diagnosed with bile duct adenoma from the year 2008 to 2022. Patients were included in this study if they 1) were ≥18 years old and 2) had pathologically confirmed bile duct adenoma. Patients who did not undergo preoperative MRI and those with concurrent cholangiocarcinoma or history of liver surgery were excluded. A total of 28 patients (with 32 bile duct adenomas overall) met the eligibility criteria. The clinical data of the patients and the imaging features of the lesions were recorded.

### Image acquisition

MRI was performed using a 3T MRI system (MAGNETOM Prisma, Siemens Healthcare, Erlangen, Germany) equipped with an 18-channel phased-array body coil. Patients were asked to abstain from solid food and water for 4–6 h before the imaging examination. The scan sequences included the following: 1) coronal T2WI (single-shot turbo spin-echo; TR = 1,400 ms; TE = 87 ms; refocusing flip angle = 150°; matrix size = 256 × 256; slice thickness/gap = 5.0/1.5 mm); 2) axial T2WI (fat-suppressed turbo-spin-echo sequence; TR = 3,000 ms; TE = 87 ms; refocusing flip angle = 100°; matrix size = 256 × 256; slice thickness 5.0 mm with 1.5 mm gap); 3) diffusion-weighted imaging (DWI; b-value = 50, 800 s·mm^-2^; TR = 5,600, TE = 58.0, refocusing flip angle = 140°; matrix size = 128 × 128); and 4) coronal and axial T1WI (Dixon sequence; TR = 3.97 ms; TE = 1.29 and 2.52 ms; flip angle = 9.0°; matrix size = 182 × 320; slice thickness = 3.0 mm with a 0.6-mm gap). In addition, dynamic T1WI was performed for 18 patients; arterial (25–40 s), portal venous (45–90 s), and delayed phase (3–5 min) images were obtained after contrast media injection (Magnevist; Schering, Berlin, Germany; 0.1 mmol/kg body weight) at a rate of 2 mL/s.

### Imaging analysis

**Figure 1:**
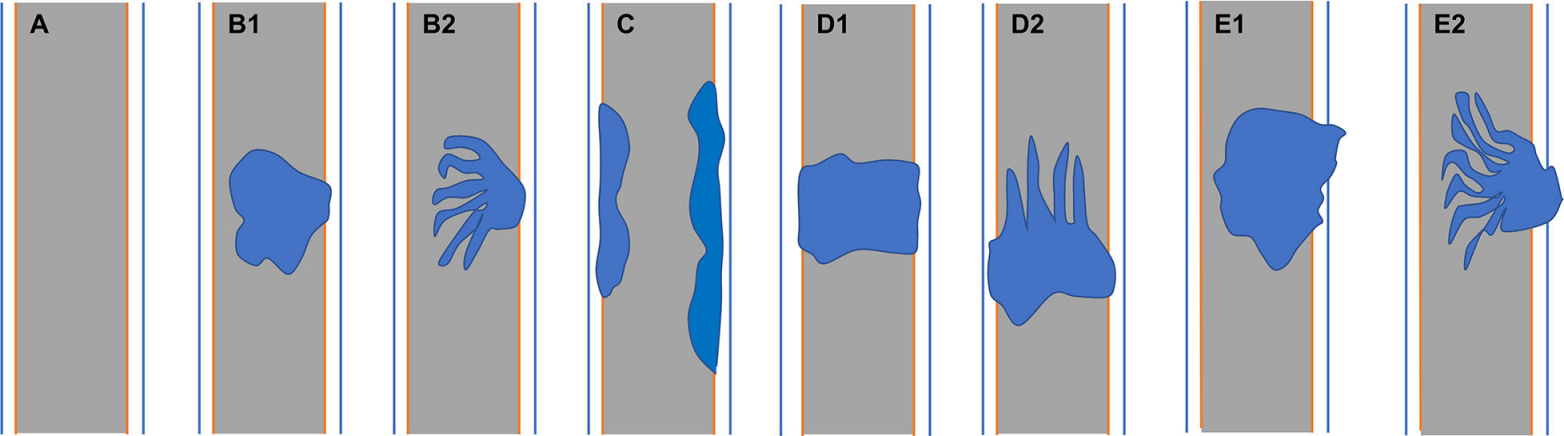
**(A)** Normal bile duct. Bile duct adenoma presents as focal eccentric-type mass **(B1, B2)**, plaque-like mass **(C)**, bile duct casting mass **(D1, D2)**, and infiltrative mass **(E1, E2)**. Frond-like superficial appearance on focal eccentric-type mass **(B2)**, bile duct casting mass **(D2)**, and infiltrative mass **(E2)**.

Two senior board-certified abdominal radiologists (with 7–10 years of experience) blinded to the clinical information retrospectively reviewed the abdominal MR images. Conclusions were arrived at by consensus. The radiologists recorded the 1) lesion location; 2) lesion morphology (focal eccentric-type mass, plaque-like mass, bile duct casting mass, or infiltrative mass, i.e., invasion of the bile duct muscle layer or growth out of the duct; **Figure 1**); 3) growth pattern; 4) signal intensity on T1WI, T2WI, and DWI (hyperintense, hypointense, or isointense as compared to the signal intensity of normal liver parenchyma); 5) enhancement degree of the adenoma in the phase with the highest degree of reinforcement (slight enhancement, moderate enhancement, or obvious enhancement as compared to that of normal hepatic parenchyma); 6) enhancement pattern (stable and persistent, wash-in and wash-out, or progressive reinforcement); and 7) appearance types of the bile duct.

The maximum size of the lesion on axial and coronal T2WI was recorded. The apparent diffusion coefficient (ADC) value of the lesion was measured at the level of its greatest extent. For ADC mapping, the region of interest (ROI) was placed to include only the solid part of the lesion, avoiding areas of liquefaction, necrosis, or bleeding as far as possible.

The diameter of the extrahepatic bile duct (EBD) was measured at the level of maximum dilatation. Dilatation was defined as 1) diameter >7 mm in patients <60 years old and without a history of cholecystectomy; 2) diameter >9 mm in patients aged ≥60 years and without a history of cholecystectomy; or 3) diameter >10 mm in patients with a history of cholecystectomy (9).

### Statistical analysis

Statistical analysis was performed with SPSS 21.0 (IBM Corp., Armonk, NY, USA). Qualitative data were expressed as percentages and compared using the chi-square test or Fisher exact test. Quantitative data were expressed as means ± standard deviation and compared using the independent-sample *t*-test. *P* < 0.05 was considered statistically significant.

## Results

### Clinical data

Out of 56 patients initially identified from the pathology department database, 28 were excluded because of incomplete preoperative imaging examinations. The remaining 28 patients (18 men, 10 women) formed the study cohort. **Table 1** shows the baseline demographic and clinical characteristics of the patients.

**Table 1:** Characteristics of the patients (n = 28) and the adenomas (n = 32).

Clinical data	Data
Age, years, mean ± SD	63.19 ± 10.72
Sex, n (%)	
Male	17 (60.71)
Female	11 (32.29)
Laboratory data, mean ± SD	
ALT (U/L)	102.96 ± 107.62
AST (U/L)	76.74 ± 67.19
ALP (U/L)	378.96 ± 344.80
GGT (U/L)	502.23 ± 549.33
Direct bilirubin (µmol/L)	89.87 ± 22.11
Indirect bilirubin (µmol/L)	24.23 ± 54.23
CA19-9 (U/mL)	1,198.96 ± 2,633.66
Pathologic findings, n (%)	
Tubular	2 (6.25)
Papillary	1 (3.13)
Tubulopapillary	29 (90.62)
Invasion of bile duct muscle layer, n (%)	
Yes	5 (15.63)
No	27 (84.38)
Clinical symptoms, n (%)	
Jaundice	12 (42.86)
Abdominal pain	6 (21.43)
Intermittent fever	5 (17.86)
Abdominal distension	1 (3.57)
Pruritus	1 (3.57)
Anorexia	1 (3.57)
Asymptomatic	4 (14.29)

SD, standard deviation; ALT, alanine transaminase; AST, aspartate transaminase; ALP, alkaline phosphatase; GGT, gamma-glutamyltransferase.

### Imaging findings

**Figure 2:**
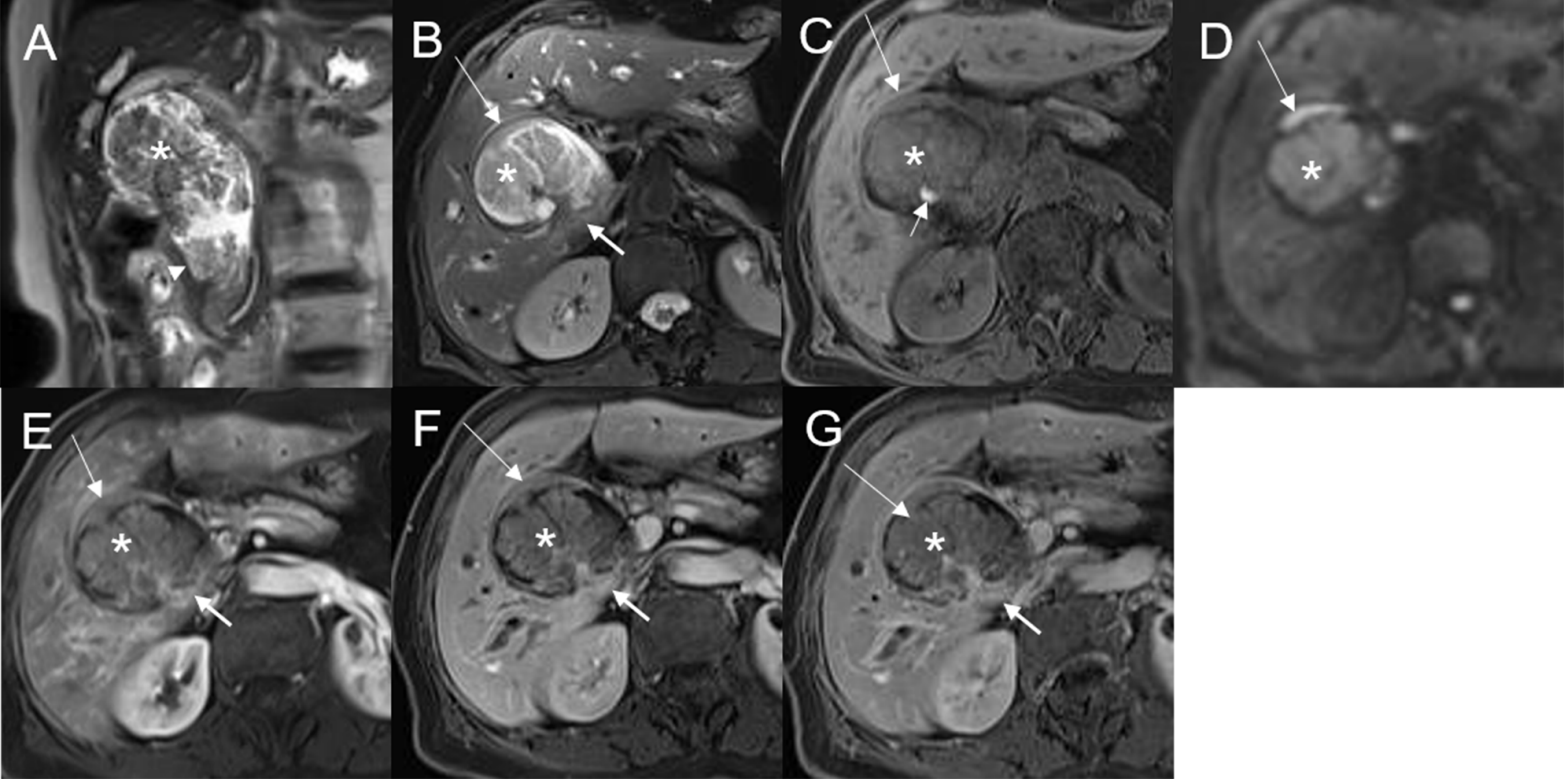
Malignant transformation of adenoma, with three lesions in the common hepatic duct and common bile duct. The first eccentric mass, with a frond-like superficial appearance (*), shows hyperintensity on T2WI **(A, B)**, hypointensity with speckled hyperintensity (short arrow) on the T1WI **(C)**, hyperintensity on DWI **(D)**, and slight continuous enhancement **(E–G)**. The second lesion, presenting as a plaque-like mass (long arrow) shows hyperintensity on T2WI **(B)**, hypointensity on the T1WI **(C)**, hyperintensity on DWI, and hyperintensity on DWI **(D)**. The T1-postcontrast arterial phase **(E)**, portal phase **(F)**, and delayed phase **(G)** all show slight enhancement. The bile duct wall is thickened at the base of the lesion (thick arrow**)**. The third lesion, presenting as an eccentric mass (arrow), is hyperintense on coronal T2WI **(A)**. The bile duct upstream and at the site of the most distal lesion is seen dilated on coronal T2WI **(A)**.

The 28 patients had a total of 32 lesions; while 26 patients had single lesions, 2 patients had three lesions each (one patient had dual plaque-like masses and an infiltrative mass, and the other patient had three plaque-like masses; **Figure 2**).

Of the lesions, 22/32 (68.75%) involved the common bile duct (CBD); while 13/32 (40.63%) lesions involved the distal CBD, 7/32 (21.88%) involved the CBD and the common hepatic duct (CHD), 2/32 (6.25%) involved only the CHD, and 1/32 (3.13%) involved only the left intrahepatic bile duct (**Table 2**). According to location, the lesions were divided into two groups: a distal CBD group (n = 13) and a non-distal CBD group (n = 19). Mean lesion size was significantly greater in the non-distal CBD group (36.91 ± 19.26 mm vs. 18.23 ± 6.80 mm, *P* = 0.001; **Table 3**).

**Table 2:** MRI characteristics of bile duct adenomas.

MRI finding	n (%)
Morphological classification	
Eccentric-type	14 (43.75)
Bile duct casting	4 (12.50)
Plaque-like	9 (28.13)
Infiltrative	5 (15.62)
Site	
CBD/distal CBD	22 (68.75)/13 (40.63)
CHD	2 (6.25)
CBD and CHD	7 (21.88)
Left hepatic duct	1 (3.13)
T1WI	
Isointensity	23 (71.88)
Hypointensity	9 (28.12)
Speckled hyperintensity	3 (9.38%)
T2WI	
Isointensity	8 (25.00)
Hyperintensity	24 (75.00)
Frond-like	8 (25.00)
Enhancement degree	
Obvious	2 (11.11)
Moderate	14 (77.80)
Slight	2 (11.11)
Enhancement pattern	
Stable and persistent	13 (72.22)
Wash-in and wash-out	5 (27.78)
Dilatation of bile duct	
Upstream of lesion	32 (100.00)
At the lesion	24 (75.00)
Downstream of lesion	6 (18.75)
Dilatation of pancreatic duct	0 (0)
DWI lesion signal	
Isointense	5 (15.63)
hyperintense	27 (84.37)
DWI signal of bile duct surrounding the lesion	
Hyperintense	2 (6.25)
Isointense	30 (93.75)
ADC, ×10^-3^mm^2^·s^-1^, mean ±SD	1.65 ± 0.38

CBD, common bile duct; CHD, common hepatic duct; T1WI, T1-weighted imaging; T2WI, T2-weighted imaging; DWI, diffusion-weighted imaging; ADC, apparent diffusion coefficient; SD, standard deviation.

**Table 3:** Demographic characteristics, laboratory findings, and MRI features of patients with adenoma in the distal CBD and non-distal CBD.

Clinical information	Distal CBD (n = 13)	Non-distal CBD (n = 19)	χ^2^/*t*		*P*	
Age, years, mean ± SD	59.15 ± 11.70	65.80 ± 9.28	-1.676		0.106
Sex						
Male	3	8	2.673		0.102
Female	10	7			
Laboratory data, mean ± SD						
ALT (U/L)	138.75 ± 102.79	70.58 ± 96.00	1.875		0.071	
AST (U/L)	90.67 ± 72.46	70.84 ± 65.77	0.786		0.438	
ALP (U/L)	344.27 ± 268.15	491.67 ± 118.66	-0.893		0.380	
GGT (U/L)	539.92 ± 598.08	478.50 ± 525.66	0.233		0.817	
TBil (µmol/L)	139.13 ± 125.33	72.04 ± 112.07	1.551		0.132	
Direct bilirubin (µmol/L)	112.39 ± 101.01	62.48 ± 109.22	1.281		0.211	
Indirect bilirubin (µmol/L)	40.49 ± 75.46	8.23 ± 9.73	1.532		0.151	
CA19-9 (U/L)	116.40 ± 102.58	1,140.63 ± 2,234.75	-1.887		0.077	
Morphological classification, n						
Eccentric-type			0.051		0.821
Positive	6	8			
Negative	7	11			
Bile duct casting					0.200
Positive	4	0			
Negative	9	19			
Infiltrative					>0.99
Positive	2	3			
Negative	11	16			
Plaque-like					0.050
Positive	1	8			
Negative	12	11			
Maximum size of lesion, mm, mean ± SD	18.23 ± 6.80	36.91 ± 19.26	-3.889		0.001
Speckled hyperintensity on T1WI, n						
Positive	1	3			0.629
Negative	12	16			
Frond-like					
Positive	1	7			0.101
Negative	12	12			
Enhancement pattern						
Stable and persistent	6	7			0.596
Wash-in and washout	1	4			
Bile duct dilatation downstream of lesion						
Positive	0	6			0.059
Negative	13	13			
Signal of lesion on DWI						
Isointense	4	1			0.132
Hyperintense	9	18			
ADC, ×10^-3^mm^2^·s^-1^, mean ± SD	1.57 ± 0.25	1.70 ± 0.44	-1.008		0.323

SD, standard deviation; ALT, alanine transaminase; AST, aspartate transaminase; ALP, alkaline phosphatase; GGT, gamma-glutamyltransferase; TBil, total bilirubin; CA19-9, carbohydrate antigen 19-9; T1WI, T1-weighted imaging; DWI, diffusion-weighted imaging; ADC, apparent diffusion coefficient.

**Figure 3:**
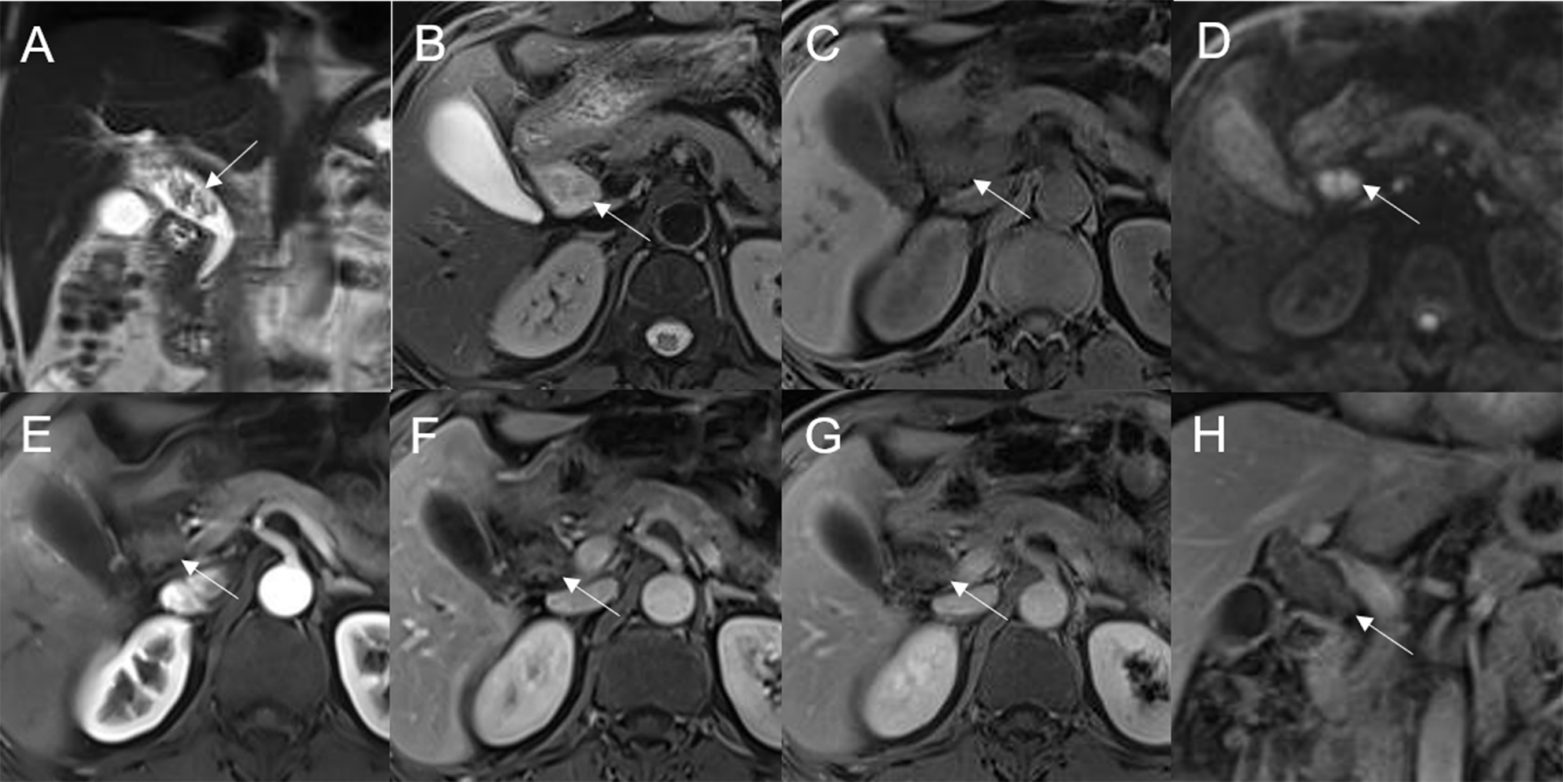
Adenoma in the common bile duct. The eccentric mass (white arrow) with a frond-like superficial appearance shows hyperintensity on T2WI **(A, B)**, hypointensity on T1WI **(C)**, and hyperintensity on DWI **(D)**. The bile duct upstream of the lesion and at the lesion is dilated on coronal T2WI **(A)**. The T1-postcontrast arterial phase **(E)**, T1-postcontrast portal phase **(F)**, and T1-postcontrast delayed phase **(G, H)** all show slight enhancement.

**Figure 4:**
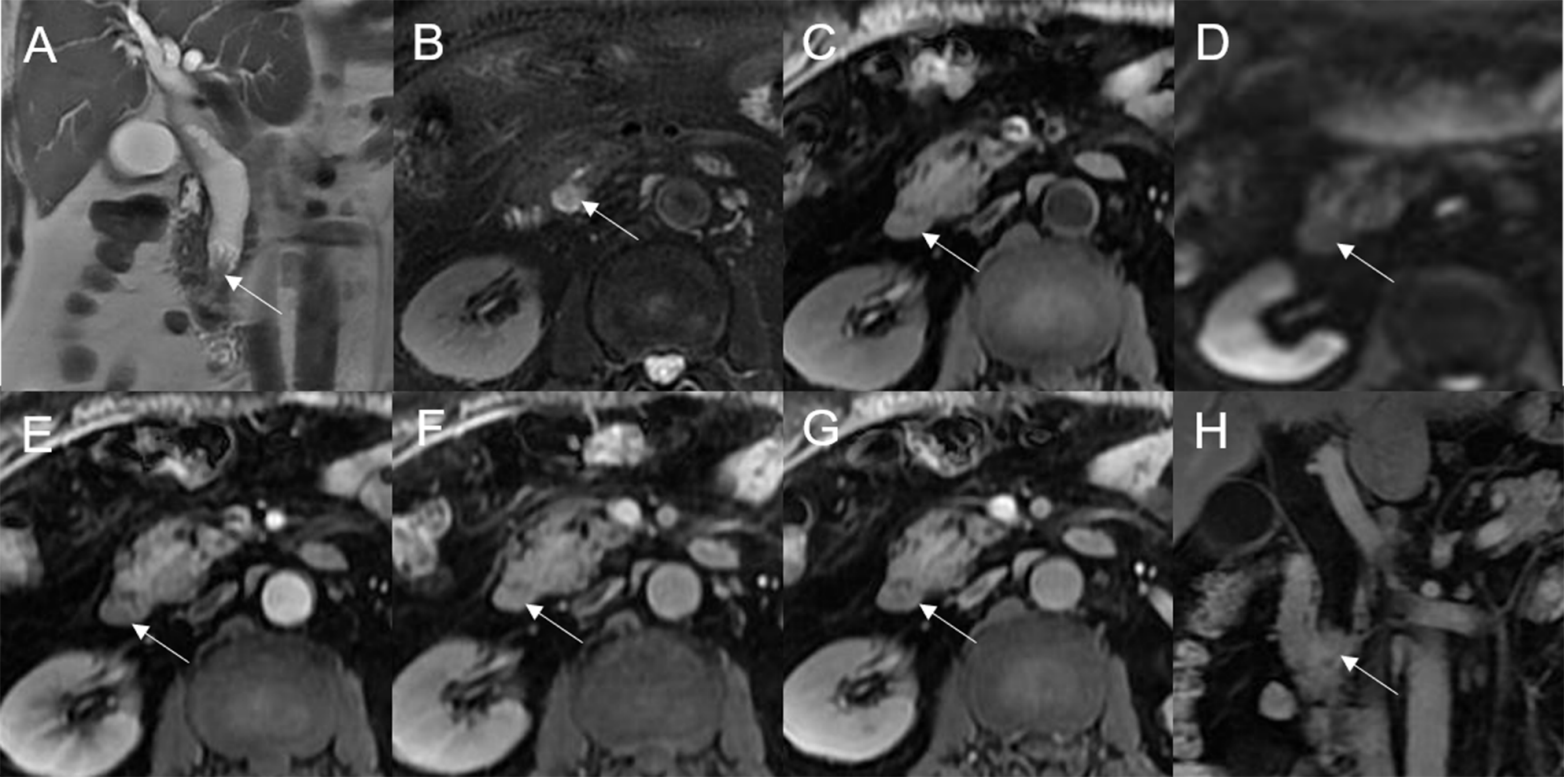
Adenoma in the distal common bile duct. The bile duct casting mass with a frond-like superficial appearance (white arrow) shows hyperintensity on T2WI **(A, B)**, isointensity on T1WI **(C)**, and hyperintensity on DWI **(D)**. There is narrowing of the bile duct lumen at the lesion site and dilation of the duct upstream of the lesion **(A)**. The T1-postcontrast arterial phase **(E)** shows slight enhancement. The T1-postcontrast portal phase **(F)** and T1-postcontrast delayed phase **(G, H)** show moderate enhancement.

**Figure 5:**
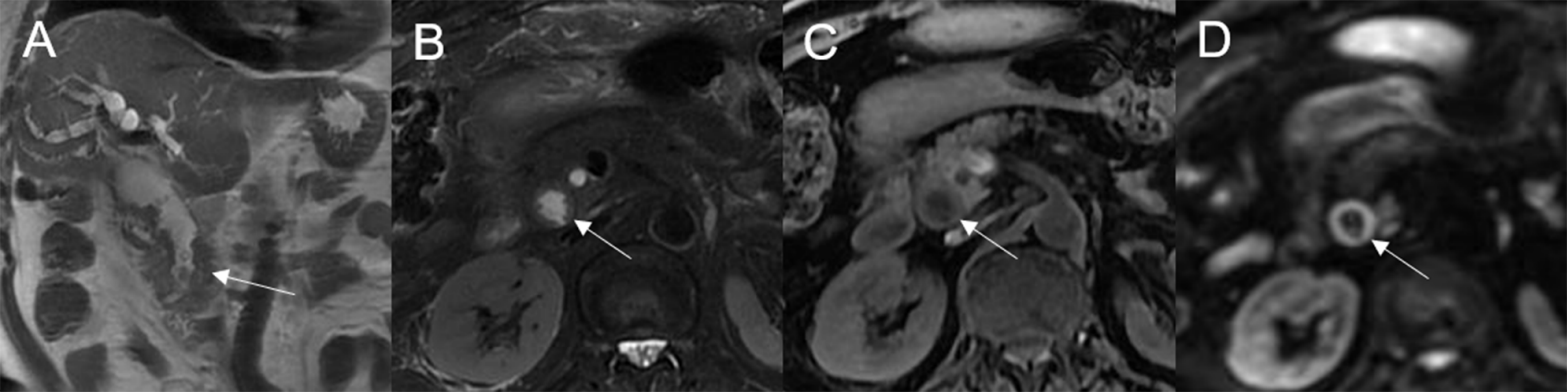
Adenoma in the common bile duct. The plaque-like mass (white arrow) shows isointensity on T2WI **(A, B)**, isointensity on T1WI **(C)**, and hyperintensity on DWI **(D)**. The bile duct lumen at the lesion site is narrow and the diameter of the bile duct is enlarged. Coronal T2WI shows dilatation of the bile duct upstream of the lesion **(A)**.

Mean lesion size in the whole sample was 30.83 ± 20.93 mm. All patients presented with an intraluminal ductal mass. While 14/32 (43.75%) lesions presented morphologically as focal eccentric-type masses (**Figure 3**), 9/32 (28.13%) presented as plaque-like masses, 4/32 (12.50%) as bile duct casting masses, and 5/32 (15.62%) as infiltrative masses. In addition, 8/32 (21.88%) lesions showed a frond-like superficial appearance accompanied by other morphologically categorized appearances other than plaque-like superficial (**Figures 4**, **5**; **Table 2**).

**Figure 6:**
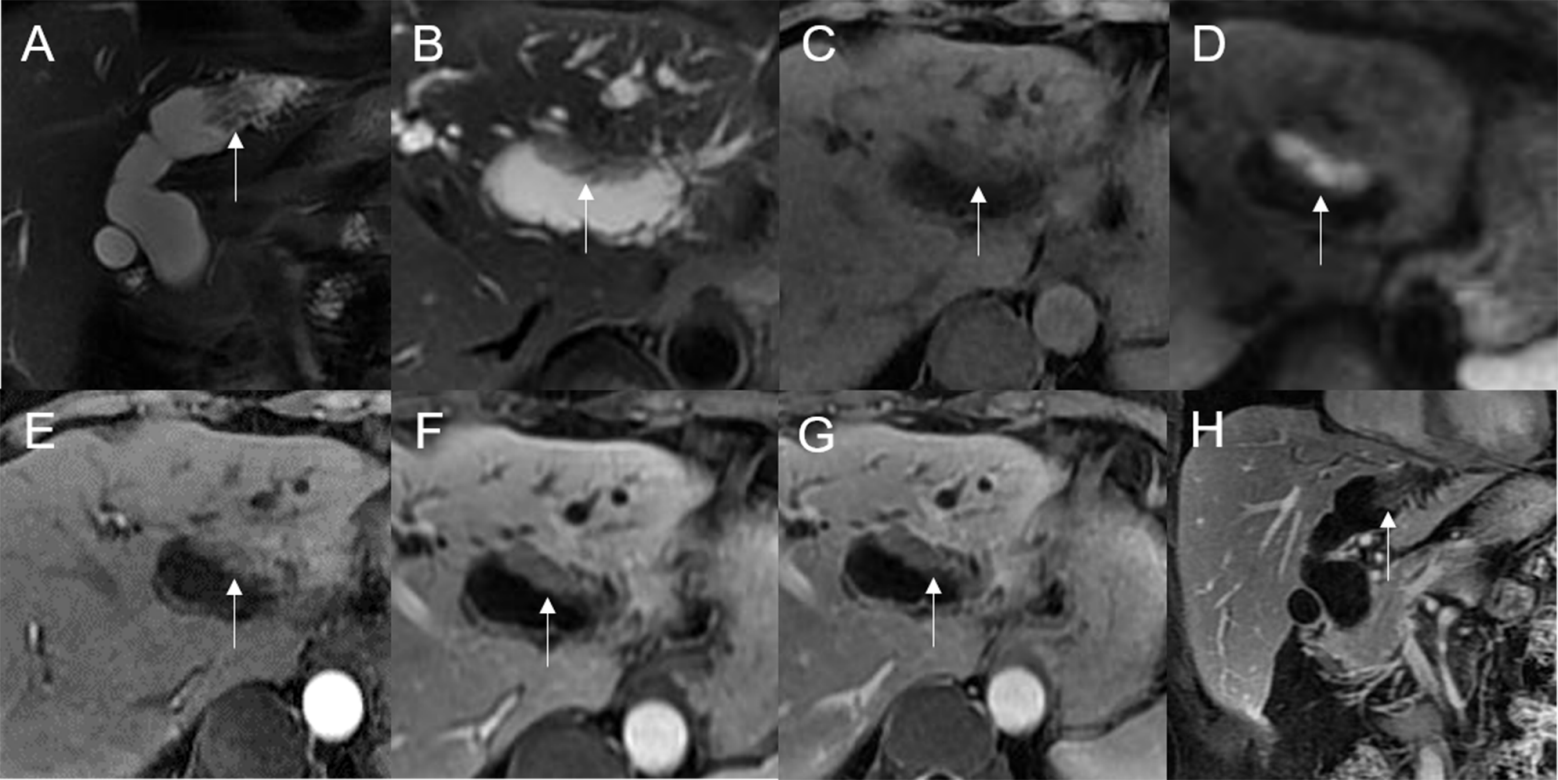
Adenoma in the left hepatic duct. The plaque-like mass (white arrow) shows isointensity on T2WI **(A, B)**, isointensity on T1WI **(C)**, and hyperintensity on DWI **(D)**. The bile duct upstream, downstream, and at the lesion is dilated on coronal T2WI **(A)**. T1-postcontrast arterial phase **(E)**, portal phase **(F)**, and delayed phase **(G, H)** all show slight enhancement.

Bile duct dilatation upstream of the lesion was present in all cases. Bile duct dilatation downstream of the lesion was present in 6/32 (18.75%) cases (**Figure 6**) and at the lesion in 24/32 (75%) cases. In 8/32 (25%) cases, there was a stricture in the bile duct lumen at the lesion, along with an enlarged bile duct diameter; while four were cases of bile duct casting masses, the other four were cases of plaque-like masses.

**Figure 7:**
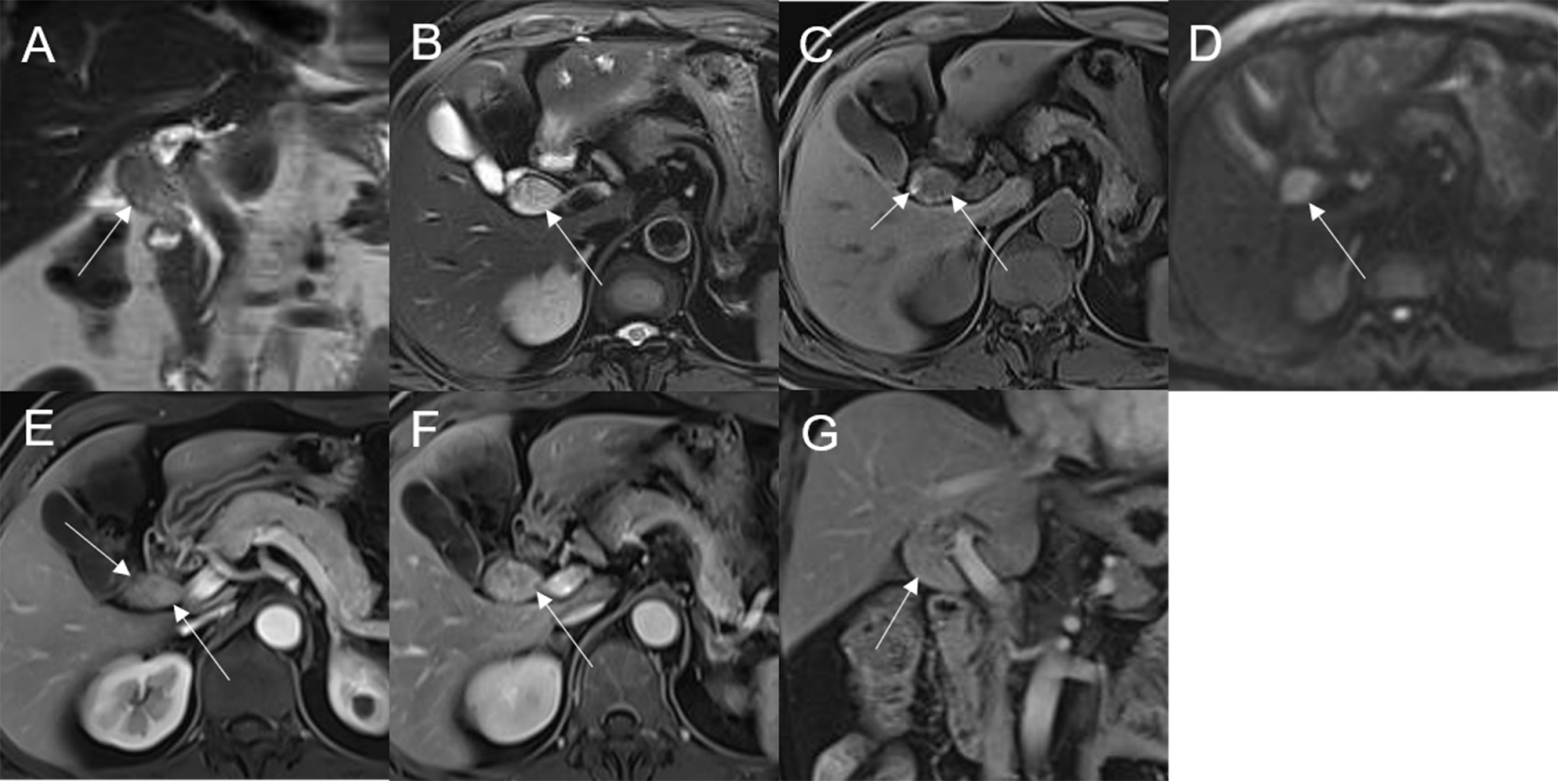
Adenoma in the common bile duct. The eccentric mass (white arrow) with a frond-like superficial appearance shows hyperintensity on T2WI **(A, B)**, hypointensity on T1WI **(C)**, and hyperintensity on DWI **(D)**. The bile duct upstream of the lesion and at the lesion is dilated on coronal T2WI **(A)**. The T1-postcontrast arterial phase **(E)** shows slight enhancement. The T1-postcontrast portal phase **(F)** and delayed phase **(G)** show moderate enhancement.

On standard MRI scanning, 24/32 (75%) lesions showed hyperintensity and 9/32 (25%) showed isointensity on T2WI, while 9/32 (28.12%) lesions showed isointensity and 23/32 (71.88%) showed hyperintensity on T1WI. In addition, 3/32 (9.38%) lesions showed speckled hyperintensity on T1WI (**Figure 7**).

On dynamic enhanced MRI, 14/18 (77.78%) lesions showed moderate enhancement, 2/18 (11.11%) showed obvious enhancement, and 2/18 (11.11%) showed slight enhancement. While 13/18 (72.22%) lesions showed stable and persistent enhancement, 5/18 (27.78%) showed wash-in and wash-out enhancement (**Table 2**).

**Figure 8:**
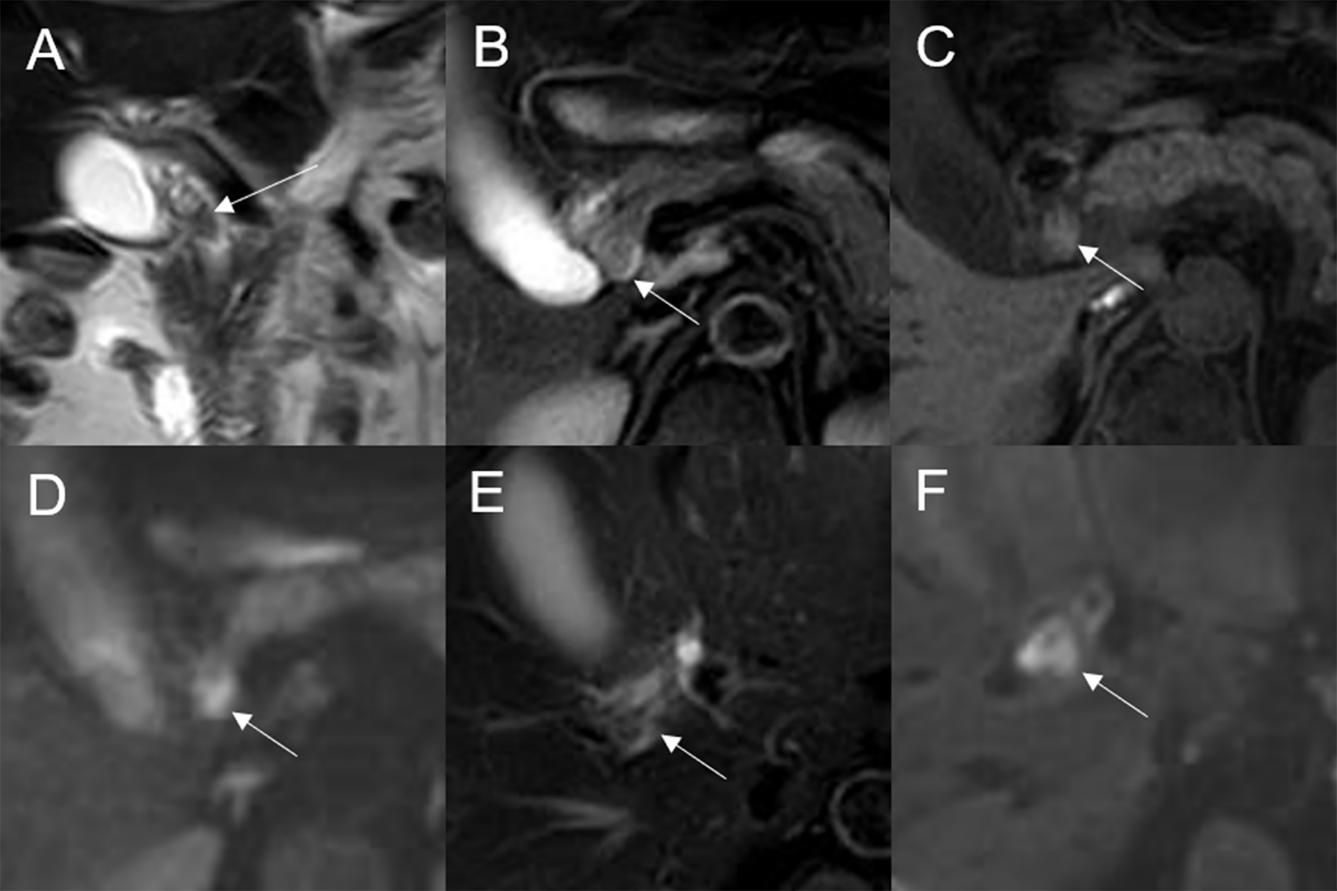
Malignant transformation of adenoma in the common bile duct. The eccentric mass with a frond-like superficial appearance (white arrow) shows hyperintensity on T2WI **(A, B)**, isointensity on T1WI **(C)**, and hyperintensity on DWI **(D)**. The bile duct wall (white arrow**)** surrounding the lesion, without any abnormal signal on T2WI **(E)**, shows hyperintensity on DWI **(F)**.

On DWI (b = 800 s·mm^-2^), 5/32 (15.63%) adenomas showed isointensity (**Figure 2**) and 27/32 (84.37%) showed hyperintensity. In 2/32 (6.25%) cases, the bile duct wall around the lesion showed hyperintensity on DWI (**Figure 8**). The mean ADC value of the lesions was 1.65 ± 0.38 × 10^-3^ mm^2^·s^-1^ (**Table 2**).

### Imaging findings in the malignant group versus benign adenoma group

According to whether or not malignant change was present, the adenomas were divided into two groups: a malignant group (n = 15) and a benign adenoma group (n = 17). Plaque-type lesions were significantly more common in the benign adenoma group, while infiltrative lesions were significantly more common in the malignant group (*P* = 0.018 and *P* = 0.015, respectively; **Table 4**).

**Table 4:** MRI findings and clinical data of malignant and benign adenomas.

Clinical information	Benign adenoma group (n = 17)	Malignant group (n = 15)	χ^2^/*t*	*P*
Age, years, mean ± SD	64.60 ± 12.55	60.54 ± 8.34	-0.991	0.331
Sex				
Male	3	8	3.743	0.053
Female	11	6		
Laboratory data				
ALT (U/L)	107.82 ± 123.09	83.79 ± 73.06	0.0642	0.526
AST (U/L)	76.47 ± 67.41	81.00 ± 71.05	-1.799	0.084
ALP (U/L)	438.29 ± 414.88	303.36 ± 224.39	-0.970	0.342
GGT (U/L)	611.38 ± 556.53	437.00 ± 566.63	-0.849	0.403
TBil (µmol/L)	111.33 ± 127.20	83.80 ± 114.46	-0.632	0.532
Direct bilirubin (µmol/L)	85.04 ± 96.74	83.04 ± 119.79	0.718	0.479
Indirect bilirubin (µmol/L)	31.93 ± 69.91	10.92 ± 8.69	1.476	0.161
CA19-9 (U/L)	816.18 ± 2,273.69	679.80 ± 1,414.76	0.195	0.847
Site				
Distal CBD	6	7	0.252	0.616
Non-distal CBD	11	8		
Maximum size of lesion	28.53 ± 21.04	30.02 ± 15.22	-0.230	0.819
Morphological classification				
Eccentric-type				
Positive	7	7	0.098	>0.990
Negative	10	8		
Bile duct casting				
Positive	2	2		>0.990
Negative	15	13		
Plaque-like				
Positive	8	1		0.018
Negative	9	14		
Infiltrative				
Positive	0	5		0.015
Negative	17	10		
Speckled hyperintensity on T1WI				
Positive	1	3		0.319
Negative	16	12		
Frond-like				
Positive	4	4		>0.990
Negative	13	11		
Enhancement pattern				
Stable and persistent	6	7		>0.990
Wash-in and washout	2	3		
Bile duct dilatation downstream of lesion				
Positive	4	2		0.659
Negative	13	13		
DWI signal of lesion				
Isointense	2	3		0.645
Hyperintense	15	12		
DWI signal of bile duct surrounding the lesion				
Hyperintense	0	2		0.480
Isointense	17	13		
ADC, ×10^-3^mm^2^·s^-1^, mean ± SD	1.77 ± 0.36	1.55 ± 0.38	-1.464	0.156

SD, standard deviation; ALT, alanine transaminase; AST, aspartate transaminase; ALP, alkaline phosphatase; GGT, gamma-glutamyltransferase; TBil, total bilirubin; CBD, common bile duct; CHD, common hepatic duct; CA19-9, carbohydrate antigen 19-9; T1WI, T1-weighted imaging; DWI, diffusion-weighted imaging; ADC, apparent diffusion coefficient.

## Discussion

The 1991 World Health Organization classification of benign bile duct tumors divides adenomas into three types: tubular adenomas, papillary adenomas, and ductal papillary adenomas (10). In our cohort, tubulopapillary adenomas were the predominant type (90.62%).

Patients with bile duct adenoma present with symptoms such as abdominal pain, jaundice, fever, dyspepsia, nausea, vomiting, fatigue, and pruritus. Pancreatitis may occur if the lesion compresses the pancreatic duct (11). Consistent with previous reports (12), we found that patients usually have elevated levels of blood bilirubin, liver enzymes, and alkaline phosphatase. In this study, the mean age at diagnosis was 63.8 years, with men being more commonly affected (male:female ratio, 1.6:1). Previous studies have reported inconsistent results on the incidence of adenoma in men versus women (13) possibly due to the low incidence of the disease and small sample sizes. Most patients have only a single lesion (92.86% in our cohort), but multiple lesions may also occur (5, 14). Only two of our patients had multiple lesions.

Bile duct adenoma may arise anywhere in the bile duct. Extrahepatic bile ducts are involved in most cases (the distal extrahepatic biliary tree was frequently involved among our patients). However, the intrahepatic bile duct, cystic duct, gallbladder, or pancreatic duct may also be involved (15). In our patients, bile duct adenoma mainly occurred in large bile ducts, including the CBD, CHD, and the left hepatic duct. The distal CBD was most commonly affected in our cohort, as has been reported in other studies (11). Lesions in the distal CBD tended to be smaller than those in the non-distal CBD probably because earlier onset of obstructive symptoms led to earlier detection. Sex distribution, laboratory results, and other imaging findings were comparable between the distal and non-distal CBD groups.

Anum et al. classified intraductal papillary neoplasm of the bile duct (IPNB) morphologically as intraluminal polypoid, patellar, invasive, or lobulated with mural nodules (8). Struyve et al. (16) reported that bile duct adenoma is accompanied by bile duct dilatation with multiple irregular filling defects. However, the expression of bile duct adenoma morphology might be incomplete due to the small number of previous cases. In our patients, bile duct adenoma presented as intraductal focal eccentric-type mass, plaque-like mass, bile duct casting mass, or infiltrative mass. Bile duct adenoma presenting as a bile duct casting mass has not been previously reported. Interestingly, we found that two growth morphologies could simultaneously occur in the same patient. There have been earlier reports of bile duct adenoma with frond-like appearance (17). In this study, the superficial frond-like appearance was seen in a few lesions (25%) other than plaque-like lesions.

In our patients, bile duct adenomas were isointense or hypointense relative to the normal liver on T1WI and isointense or hyperintense on T2WI. Among the 32 lesions studied, most showed isointensity on T1WI and hyperintensity on T2WI. Speckled hyperintensity on T1WI was seen in three lesions, probably indicating intralesional hemorrhage. After contrast injection, most lesions showed moderate enhancement, but other levels of enhancement were seen in a few. Stable and persistent enhancement was most common, but wash-in and wash-out pattern was also seen in some cases. These findings are consistent with previous reports (18). On DWI, most lesions were hyperintense, but other signal intensities were also seen.

In a previous imaging-based study, 60% of patients with extrahepatic bile duct adenoma had bile duct dilatation (11); however, the authors did not provide a detailed description of features of the dilated duct. In this study, bile duct dilatation was seen upstream of the lesion in all cases; while dilatation at the lesion site was common, dilatation downstream of the lesion was seen in only a few cases. Among the six cases with downstream bile duct dilatation, two lesions were secreting mucus with a doughy or jelly-like consistency, which may have blocked the distal bile duct. There have been previous reports of this phenomenon in patients with IPNB but not in patients with bile duct adenoma (19). Four of the six cases of downstream dilatation were in patients with multiple adenomas, with the dilatation being due to blockage of the bile duct by the distal lesion. Stricture of the bile duct lumen at the lesion site, with overall increase in bile duct diameter and upstream dilatation, was seen in all cases of bile duct casting masses and several cases of plaque-like masses. As mentioned earlier, in cases of plaque-like masses, bile duct dilatation at the lesion site was because of duct obstruction by lesions or secretions downstream.

Bile duct adenomas are benign epithelial tumors with potential for malignant transformation (2). In our study, malignant transformation was identified in 15 of the 32 lesions. Age, sex, and laboratory data were not significantly associated with malignant transformation. In the absence of specific clinical features or laboratory parameters, diagnosis of malignancy can be challenging. A previous study reported that an elevation in carbohydrate antigen 19-9 (CA19-9) may be indicative of malignant transformation of adenoma (20). However, we found no significant difference in CA19-9 between the malignant group and the benign adenoma group in our study; this may have been due to the limited number of cases. The value of CA19-9 for the diagnosis of malignant transformation needs further exploration.

Previous studies have found that large gastrointestinal adenomas are easier to manage than smaller ones (21). However, in this study, we found no significant difference in lesion size between the malignant group and the benign adenoma group. This may have been because the small size of the bile duct lumen results in early clinical symptoms, with detection of the tumor before it becomes large. Plaque-like masses were more common in the benign adenoma group, while infiltrative masses were seen only in the malignant group. Thus, infiltrative behavior may indicate malignant transformation. In two cases in this study, the bile duct wall surrounding the lesion showed hyperintensity on DWI, and pathology confirmed malignant change in the lesion with invasion of the bile duct. This sign was present only in the malignant group. However, there was no significant difference in this sign between the malignant group and the benign adenoma group. Additionally, the other signal characteristics, dilated shape of the bile duct, DWI, enhancement degree, and mode of bile duct adenoma development have limited ability to distinguish malignant transformation of adenoma.

In the clinic, bile duct adenoma has to be distinguished from cholangiocarcinoma and hepatocellular carcinoma (HCC). Cholangiocarcinoma presents as a mass-forming, periductal-infiltrating, and intraductal-growing lesion, often accompanied by upstream dilatation of the bile duct. Frond-like superficial appearance and dilatation downstream of the lesion are not seen. The periductal-infiltrating type of cholangiocarcinoma presents with thickening of the bile duct wall and narrowing of the bile duct lumen at the lesion site. The intraductal-growing type of cholangiocarcinoma is relatively rare; it presents as a papillary tumor with dilatation of the duct at the lesion site and may be difficult to differentiate from the eccentric-type bile duct adenoma. The mass-forming type of cholangiocarcinoma presents as an extramural growth in the bile duct area; the bile duct within the lesion is not clearly displayed on imaging. In addition, intrahepatic cholangiocarcinoma may be accompanied by hepatic capsular retraction and targetoid appearance (22). Meanwhile, HCC usually occurs in the background of liver cirrhosis and is typically accompanied by elevated serum alpha-fetoprotein level. The tumor avidly enhances in the arterial phase, with “wash-out” and/or capsule appearance in the portal venous or delayed phase. It rarely involves bile ducts, so bile duct dilatation is not often seen (23). In comparison, bile duct adenoma mostly occurs in large bile ducts; intrahepatic bile duct adenomas are relatively uncommon. It displays intraductal growth, sometimes with a frond-like superficial appearance, and may be associated with bile duct dilatation at the lesion site or upstream and/or downstream of the lesion.

This study has several limitations. First, this was a retrospective analysis of data from a single center; therefore, selection bias cannot be ruled out. Second, the differences in MRI features between various pathological types could not be investigated due to limited pathological data. Third, few patients had postoperative follow-up imaging, so there were limited data on recurrence and any further treatment delivered. Finally, CT examination was not routinely performed for the patients; therefore, the CT manifestations of bile duct adenoma and the combined efficacy of CT and MRI were not analyzed.

## Conclusion

Bile duct adenoma most commonly occurs in the distal common bile duct, usually showing intraductal growth and isointensity on T1WI and hyperintensity on T2WI, with mostly stable, persistent, and moderate enhancement. These tumors usually show hyperintensity on DWI and are associated with bile duct dilatation upstream of the lesion. In addition, bile duct dilatation downstream of the lesion may be a feature of bile duct adenoma. Adenoma presenting as an infiltrative mass or with DWI hyperintensity of the bile duct wall surrounding the lesion may be an indication of malignant transformation.

## Data availability statement

The raw data presented in this article are not readily available because of institutional restrictions. Requests to access the datasets should be directed to JL, liujingjing198631@126.com.

## Ethics statement

The studies involving humans were approved by the Ethics Committee of the First Affiliated Hospital of Zhengzhou University. The studies were conducted in accordance with the local legislation and institutional requirements. The ethics committee/institutional review board waived the requirement of written informed consent for participation from the participants or the participants’ legal guardians/next of kin because this study belongs to retrospective analysis.

## Author contributions

Guarantor of integrity of the entire study: MYH and JL. Study concepts and design: JC and XG. Literature research: MYH, CXL and YZ. Data collection: MYH and MNH. Data analysis: MYH, CXL and MNH. Article preparation: MYH. Article review: MYH, JL, JZ, CXL and YZ. All authors read and approved the final version of the article.
